# Supplementary material for: Modulation of Gut Microbiota through Low-Calorie and Two-Phase Diets in Obese Individuals
Source: Nutrients. 2023 Apr 11;15(8):1841. doi: 10.3390/nu15081841 (PMC10140827; doi:10.3390/nu15081841)
Supplement: Supplementary file 1 [file nutrients-15-01841-s001.zip › Table S1.pdf]

Table S1. Daily meal plain in the low-calorie and two-phase diets.

Diet	*Breakfast*	*Snack*	*Lunch*	*Snack*	*Dinner*
Low-calorie	200 ml of milk or tea with 2 slices of crispy toast (toast francais) with jam and no added sugar	Dried fruit (10 almonds or 10 hazelnuts or 5 walnuts or 8 cashews)	70 g of rice or spelled with vegetables or sauce plus tuna or 80 g of salmon	Seasonal fruit	140 g of grilled turkey breast or 110 g of pork steak, 200 g of vegetables, 70 g of wholemeal or rye bread
First phase – ketogenic	Bullet Coconut Coffee (LightFlow)	40 gr of Mini Carrè (LightFlow) with a veil of fruit cream	90 gr of pasta with vegetables or mushrooms with salad or grilled radicchio	Bullet Coconut Coffee (LightFlow)	200 gr of grilled salmon or swordfish with cooked vegetables, 40 gr of Mini Carrè (LightFlow)
Second phase – low-calorie	200 ml of milk or tea with 2 slices of crispy toast (toast francais) or 2 slices of Mini Carrè (LightFlow) with jam, Coconut coffee (CarboLight)	Dried fruit (10 almonds or 10 hazelnuts or 5 walnuts or 8 cashews)	70 g of rice or 1 portion of pasta (LighFlow) with vegetables or sauce plus tuna or salmon, 100 gr of light mozzarella or 120 gr of ricotta	Seasonal fruit	160 g of grilled turkey breast or 130 g of pork steak, 200 g of vegetables, 70 g of wholemeal or rye bread or 2 slices of Mini Carrè (LightFlow)
