# Supplementary material for: Modulation of Gut Microbiota through Low-Calorie and Two-Phase Diets in Obese Individuals
Source: Nutrients. 2023 Apr 11;15(8):1841. doi: 10.3390/nu15081841 (PMC10140827; doi:10.3390/nu15081841)
Supplement: Supplementary file 1 [file nutrients-15-01841-s001.zip › Table S2.pdf]

Table S2. Anthropometric characteristics of the participants before (baseline) and after low-calorie and two-phase diets.

Patients		Height (cm)	Weight (kg)		BMI		Abdominal circumference		Fat mass		Muscular mass	
			Baseline	Low-calorie diet	Baseline	Low-calorie diet	Baseline	Low-calorie diet	Baseline	Low-calorie diet	Baseline	Low-calorie diet
COD A01	F	1,6	75,00	73,00	29,30	28,52	112,00	111,00	28,10	27,60	31,00	30,40
COD A02	F	1,49	64,00	60,40	28,83	27,21	101,00	99,00	19,00	17,00	30,00	28,30
COD A03	F	1,58	92,80	88,00	37,17	35,25	132,00	124,00	44,70	42,00	31,00	29,40
COD A04	M	1,71	101,00	95,30	34,54	32,59	114,00	110,00	25,40	20,90	52,20	51,00
COD A05	F	1,58	75,3	73,00	30,16	29,24	112,00	106,00	28,30	26,70	30,90	30,00
COD A06	M	1,77	96,20	95,30	30,71	30,42	114,00	110,00	20,30	19,90	59,70	59,20
COD A07	F	1,7	155,00	150,00	53,63	51,90	147,00	145,00	85,50	83,50	45,70	42,20
COD A08	F	1,58	89,70	86,30	35,93	34,57	112,00	107,00	36,30	33,70	36,70	36,50
COD A09	F	1,58	88,00	88,00	35,25	35,25	104,00	104,00	36,20	36,20	33,40	33,30
COD A10	F	1,67	120,00	118,00	43,03	42,31	126,00	124,00	57,70	56,10	41,80	40,70
COD A11	F	1,58	106,00	97,00	42,46	38,86	124,00	120,00	48,00	42,90	40,00	37,80
COD A12	F	1,64	91,00	84,50	33,83	31,42	112,00	105,00	33,70	29,20	40,70	40,70
COD A13	F	1,55	81,20	76,20	33,80	31,72	104,00	100,00	28,30	24,20	41,10	40,20
COD A14	F	1,6	73,00	68,20	28,52	26,64	103,00	93,00	28,80	24,00	30,30	30,40
COD A15	F	1,59	75,30	70,20	29,79	27,77	101,00	89,00	22,80	20,00	38,70	37,90
COD A16	M	1,8	87,50	81,90	27,01	25,28	89,00	88,00	20,10	15,60	46,20	45,50
COD A17	F	1,6	103,60	97,70	40,47	38,16	125,00	120,00	38,90	36,50	50,70	46,00
COD A18	F	1,55	73,50	67,20	30,59	27,97	100,00	98,00	22,40	17,40	40,90	40,50
COD A19	F	1,65	80,90	71,80	29,72	26,37	110,00	100,00	29,80	24,10	36,20	34,20

Patients		Height (cm)	Weight (kg)		BMI		Abdominal circumference		Fat mass		Muscular mass	
			Baseline	Two-phase diet	Baseline	Two-phase diet	Baseline	Two-phase diet	Baseline	Two-phase diet	Baseline	Two-phase diet
COD B01	M	1,65	91,50	84,40	33,61	31,00	119,00	108,00	28,90	21,90	41,00	43,10
COD B02	M	1,70	104,50	98,00	36,16	33,91	122,00	114,00	28,30	22,80	56,20	58,70
COD B03	F	1,59	66,80	64,20	26,42	25,39	98,00	94,00	22,00	19,60	31,40	31,90
COD B04	F	1,60	99,10	91,4	38,71	35,70	105,00	100,00	37,20	31,70	41,20	40,00
COD B05	M	1,74	105,50	94,50	34,85	31,21	112,00	101,00	31,20	24,00	54,00	49,90

COD B06	M	1,86	102,50	95,50	29,63	27,60	112,00	106,00	28,50	26,60	53,40	52,00
COD B07	F	1,69	76,60	71,50	26,82	25,03	111,00	99,00	27,40	24,30	32,00	31,50
COD B08	F	1,66	97,90	90,70	35,53	32,91	109,00	102,00	39,40	36,00	42,00	41,00
COD B09	F	1,65	108,90	103,50	40,00	38,02	117,00	106,00	37,40	32,00	61,40	61,00
COD B10	F	1,60	112,80	104,20	44,06	40,70	136,00	124,00	53,00	46,10	39,60	38,90
COD B11	M	1,78	116,80	106,60	36,86	33,64	124,00	114,00	35,80	30,00	58,60	57,10
COD B12	F	1,60	73,00	70,00	28,52	27,34	111,00	108,00	27,60	25,40	30,40	30,10
COD B13	F	1,58	98,20	93,20	39,34	37,33	120,00	112,00	42,90	40,00	35,00	35,50
COD B14	F	1,55	76,10	69,00	31,68	28,72	100,00	94,00	23,10	16,90	41,40	40,70
COD B15	F	1,55	67,20	59,80	27,97	24,89	95,00	90,00	16,90	10,00	41,70	40,10
COD B16	F	1,67	80,10	74,20	28,72	26,61	107,00	103,00	26,20	22,30	40,00	38,10
COD B17	F	1,73	96,90	90,00	32,38	30,07	122,00	115,00	38,30	32,70	40,20	38,00
COD B18	F	1,70	74,90	65,90	25,92	22,80	106,00	100,00	22,50	16,30	35,70	34,70
COD B19	F	1,67	120,00	112,00	43,03	40,16	130,00	120,00	56,10	52,00	41,10	40,90
