# Supplementary material for: Modulation of Gut Microbiota through Low-Calorie and Two-Phase Diets in Obese Individuals
Source: Nutrients. 2023 Apr 11;15(8):1841. doi: 10.3390/nu15081841 (PMC10140827; doi:10.3390/nu15081841)
Supplement: Supplementary file 1 [file nutrients-15-01841-s001.zip › Table S3.pdf]

Table S3. Clinical characteristics of the participants before (baseline) and after low-calorie and two-phase diets.

Patients	Glucose		Cholesterol		Tryglicerid		Insulin		Cortisol		Glycated hemoglobin	
	Baseline	Low-calorie diet	Baseline	Low-calorie diet	Baseline	Low-calorie diet	Baseline	Low-calorie diet	Baseline	Low-calorie diet	Baseline	Low-calorie diet
COD A01	114	108	179	209	122	125	12,89	10,14	26,3	21,4	5,4	6,1
COD A02	78	80	234	227	105	68	5,52	4,44	11,8	17,7	5	5,5
COD A03	88	91	240	281	147	124	12,63	6,58	5,7	11	5,3	5,2
COD A04	112	106	165	190	172	144	19,64	17	7,9	13,4	5,5	5,5
COD A05	102	94	144	172	90	62	4,29	7,89	9,4	18,2	5,1	5,4
COD A06	101	94	155	169	120	102	14,34	12,16	16,2	12	5	5,3
COD A07	150	156	188	238	84	123	69,81	107,06	10,9	10,7	6	7,3
COD A08	101	88	175	209	159	201	12,22	9,62	9,4	9,3	5,7	6,7
COD A09	89	82	177	179	40	41	4,89	2,26	10,2	12,7	5,9	5,5
COD A10	88	93	182	167	79	79	13,94	15,56	13,1	9,3	5,4	5,8
COD A11	102	95	212	187	100	107	23,34	12,33	24,2	17,2	6,1	5,1
COD A12	89	91	221	186	113	61	8,7	6,89	23,4	14,8	5,8	5,6
COD A13	106	108	205	240	111	160	42,49	52,42	12,8	14,6	5,7	5,9
COD A14	98	88	120	164	49	69	9,15	6,96	30,6	20,8	5,4	5,5
COD A15	84	85	210	196	128	119	4,07	4,54	8,6	15,7	5,4	5,4
COD A16	79	82	186	149	75	58	7,11	4,02	10	15,4	5,9	5
COD A17	135	87	153	137	160	124	14,46	9,01	15,6	14,3	7,6	6,8
COD A18	95	85	209	200	101	88	11,29	6,36	11,4	11,4	5,9	5,5
COD A19	118	114	145	128	252	168	14,63	10,49	13,8	10	7,3	6,3

Patients	Glucose		Cholesterol		Tryglicerid		Insulin		Cortisol		Glycated hemoglobin	
	Baseline	Two-phase diet	Baseline	Two-phase diet	Baseline	Two-phase diet	Baseline	Two-phase diet	Baseline	Two-phase diet	Baseline	Two-phase diet
COD B01	107	101	200	250	262	244	14,87	6,85	10,1	19,6	6	5,7
COD B02	98	99	193	212	110	126	17,81	9,43	13,4	9,4	5,7	5,6
COD B03	77	98	219	210	123	82	5,88	8,59	22,2	13,8	5,6	6,1
COD B04	91	93	166	210	130	196	35,41	36,63	13,6	20,5	5,3	5,4
COD B05	107	99	225	200	103	78	12,68	7,17	12	7	5,6	4,9

COD B06	86	93	170	163	98	78	14,46	10,84	20	18,9	5,3	5,5
COD B07	81	87	267	240	158	103	5,04	5,67	15,4	18,5	6,3	5,8
COD B08	91	62	172	138	113	80	16,74	4,83	21,9	19,5	5,2	4,9
COD B09	107	92	210	235	153	170	17,14	14,5	14,2	14,4	4,4	6,1
COD B10	93	96	220	231	130	78	9,23	7,5	13,8	13,4	4,8	5,9
COD B11	98	103	138	138	91	92	36,09	56,29	14,8	11,7	5,7	5,7
COD B12	102	102	200	199	104	104	8,54	4,39	11,5	17,3	6,4	6
COD B13	108	95	199	194	106	93	19,38	7,74	18	16,6	6	6,3
COD B14	92	83	221	174	119	98	8,35	3,8	12,5	6,1	5,5	5,5
COD B15	85	76	200	181	88	66	6,36	2,28	11,4	12,9	5,5	5,4
COD B16	98	105	191	158	40	49	20,18	15,6	14,2	4	5,2	5,4
COD B17	96	88	270	230	87	116	15,6	9,24	9,2	10,4	5,6	6,5
COD B18	84	105	244	267	98	95	8,78	12,9	20,1	15,2	5,4	5,6
COD B19	95	87	159	145	96	65	9,26	3,89	11,9	14,3	4,8	4,6
